# Re-engagement in HIV care following a missed visit in rural Uganda

**DOI:** 10.1186/s13104-018-3865-9

**Published:** 2018-10-25

**Authors:** Maria Sarah Nabaggala, Rosalind Parkes-Ratanshi, Ronnie Kasirye, Agnes Kiragga, Barbara Castlenuovo, Ian Ochaka, Lilian Nakakawa, Diana Asiimwe Bena, Andrew Mujugira

**Affiliations:** 1 Infectious Diseases Institute, College of Health Sciences, Makerere University, Kampala, Uganda; 2 Cambridge Institute of Public Health, University of Cambridge, Cambridge, UK; 3 Department of Epidemiology and Biostatistics, College of Health Sciences, Makerere University, Kampala, Uganda

**Keywords:** HIV, ART, Retention, PLHIV tracking, Return to care

## Abstract

**Objective:**

We conducted a retrospective cohort study to assess the effect of tracking People Living with HIV (PLHIV) after missed clinic visits and factors associated with return to care in rural Uganda. We assessed retention in care among 650 HIV-infected women and men. We used univariable and multivariable generalized linear models to assess demographic and self-reported factors associated with re-engagement in HIV care.

**Results:**

Of 381 PLHIV who ever missed a scheduled appointment, 68% were female and most (80%) had initiated ART. Most (70%) of those tracked returned to care. Relative to men, women (adjusted risk ratio [ARR] 1.23; 95% confidence interval (CI) 1.05–1.43; p = 0.009) were more likely to return to care after active tracking. PLHIV who missed scheduled visits for other reasons (forgetting, adequate drug supplies, or long distance to clinic) had reduced odds of return to care (ARR 0.41; 95% CI 0.28–0.59; p < 0.001). These data support close monitoring of patient retention in HIV care and active measures to re-engage those who miss an appointment. Furthermore, they highlight the need for targeted interventions to those more resistant to re-engagement such as men.

## Introduction

Uganda is one of 15 countries that accounted for 75% of the 2.1 million new HIV infections that occurred in 2015 [1]. Uganda has scaled up coverage of antiretroviral therapy (ART), which durably suppresses plasma and genital tract viral load, decreases HIV-associated mortality, and prevents HIV transmission [2, 3]. The proportion of HIV-infected adults receiving ART in Uganda increased from an estimated 37–57% in 2011 to 81% in 2016/2017 [4]. Increased ART coverage was accompanied by a 51% decrease in the number of new HIV infections and a 45% reduction in HIV-related mortality between 2010 and 2016 [5].

Patient retention in HIV care is key to achieving Joint United Nations Programme on HIV/AIDS (UNAIDS) and national 90-90-90 targets: 90% of all people with HIV to be diagnosed and know their status, 90% of all HIV-infected people to receive ART, and 90% of persons receiving ART to be virally suppressed by 2020 [6, 7]. Continuous engagement with the healthcare system by HIV infected individuals is a key challenge for HIV treatment programs [8]; losses occur at each step of the HIV care cascade [9], and most attrition occurs during the first 24 months of ART [10]. Despite the personal and public health benefits of HIV treatment, global retention in care on ART at 12 months was approximately 74% in 2016 [11]. In Uganda, attrition rates of up to 20% have been reported 12 months after ART initiation [12].

Persons not retained in care experience treatment interruptions and viral non-suppression, which increases risk of drug resistant virus, and compromises personal and population-level benefits of ART [11]. Although no uniform definition of retention in care exists, at least one visit to an HIV care provider every 6 months is typically used to allow for clinically stable and virologically suppressed PLHIV who require fewer provider interactions [13–17]. Strategies to improve retention in HIV care include peer support, targeted counselling, mobile phone tracking and home visits [13]. Targeted retention interventions could minimize ART interruptions and reduce HIV-related morbidity and mortality [11]. We undertook a study at a rural government hospital in North-Eastern Uganda, with specific challenges of an agro-pastoralist community which practices semi-nomadic livestock rearing and whose settlement patterns are determined by availability of pasture and water sources [18]. We assessed the effect of patient tracking using phone calls and/or home visits on return to care among PLHIV who missed their scheduled visits.

## Main text

### Methods

#### Population and procedures

**Fig. 1:**
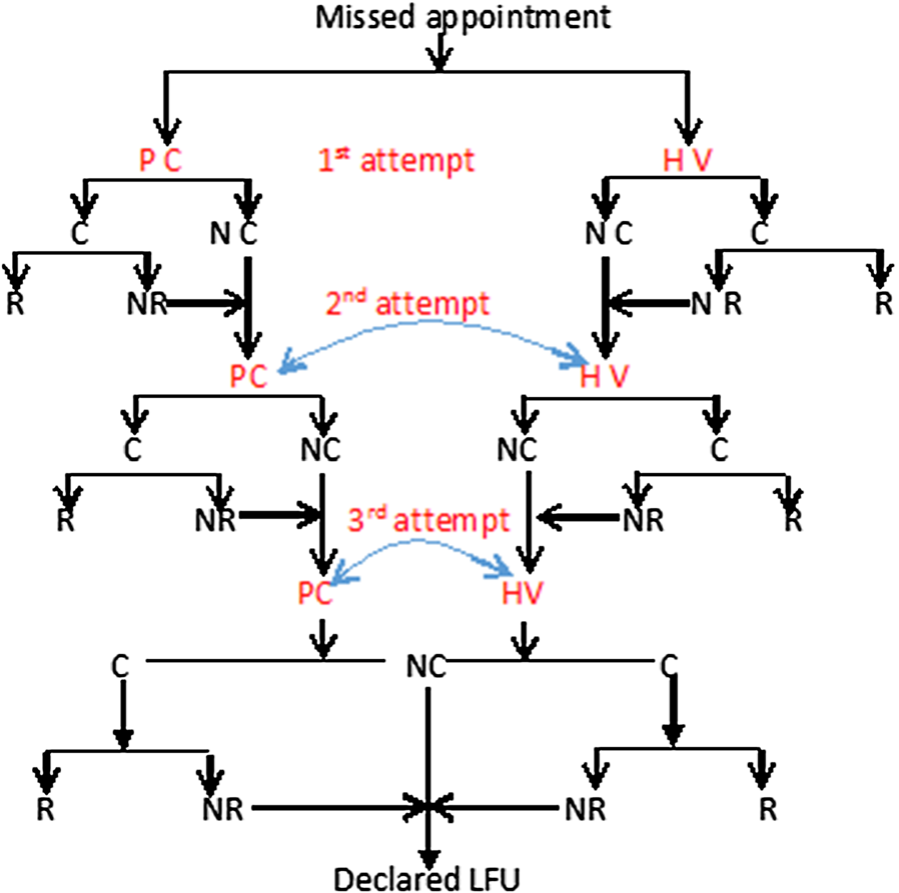
PLHIV contact (phone call and/or home visit) flow diagram. PC, phone call; HV, home visit; C, contacted; NC, not contacted; R, returned; NR, not returned; LTFU, lost to follow up

Between January 2014 and August 2015, we conducted a retrospective cohort study of PLHIV receiving HIV care at Moroto Regional Referral Hospital (RRH). The HIV program at this facility was supported by the Civil Society Fund Regional Referral Hospitals Project [19]. We included in the study PLHIV who enrolled in the ART clinic during the study period, missed a scheduled visit, and had medical records in the Open Medical Records System (OpenMRS^®^) database. Those who missed a scheduled clinic visit were contacted by telephone after 5 days (Fig. 1). The clinic had a designated counsellor to actively track persons who missed one or more visits. If the PLHIV had no telephone contact or failed to return to the clinic after several calls, a home visit was conducted by clinic staff, an expert client or a peer leader. PLHIV were tracked for 3 consecutive months after a missed scheduled visit. Those who did not return were flagged as lost to follow-up. These active tracking measures were intended to encourage PLHIV to return to active follow-up, address psychosocial issues and better understand circumstances that led to failure to turn up for the scheduled clinic visit. Data on PLHIV age, gender, ART status, tracking method, return to clinic, and reason(s) for missed visits were collected on PLHIV tracking/follow-up forms.

#### Statistical analysis

Return to care (the primary outcome) was defined as return to care within 3 months of being tracked. Descriptive statistics and frequency distributions were performed for continuous and categorical data, respectively. To identify factors associated with returning to care after active tracking, we categorized PLHIV who were tracked after missing an appointment by whether they did or did not return to care. A generalized linear model was used to estimate risk ratios of return to care after adjusting for underlying differences in predictor variables (age, gender, ART status, mode of contact and reason(s) for missing scheduled clinic visit). Significance was set at p < 0.05. Data were analyzed using Stata version 14 (StataCorp LLC, College Station, Texas).

### Results

Of the 650 persons in HIV care during the study period, 381 (59%) had ever missed a scheduled clinic visit and were included in the present analysis. For these 381, the median age was 30 years [interquartile range (IQR), 23–35], 259 (68%) were female and 306 (80%) had initiated ART (Table 1). Overall, 598 phone calls and 472 home visits were conducted during the study period.

**Table 1:** Distribution of patient characteristics stratified by return to HIV care and predictors of return to care having missed a scheduled appointment after tracking

Characteristic N (%) or median (IQR)	Did not return to HIV care (n = 114)	Returned to HIV care (n = 267)	p-value	Adjusted OR (95% CI)	p-value
Age in years, median (IQR)	30 (25–36)	29 (22–34)	0.01	0.99 (0.98–1.01)	0.265
Gender					
Male	53 (43.4)	69 (56.6)	< 0.001	Reference	
Female	61 (23.5)	198 (76.5)		1.23 (1.05–1.43)	0.009
ART status					
ART naive	33 (44.0)	42 (56.0)	0.003	Reference	
On ART	81 (26.5)	225 (73.5)		1.16 (0.96–1.39)	0.119
Tracking method					
Phone call	63 (26.7)	173 (73.3)	0.08	Reference	
Home visit	51 (35.2)	94 (64.8)		0.98 (0.87–1.11)	0.832
Reason for missing appointment					
Travelled	51 (31.5)	111 (68.5)	< 0.001	Reference	
Unavailable	38 (67.9)	18 (32.1)		0.85 (0.75–0.95)	0.006
Other	25 (15.3)	138 (84.7)		0.41 (0.28–0.59)	< 0.001

Seventy percent of PLHIV tracked by phone call and/or home visits returned to care. Of these, 74% were women. PLHIV were as likely to return to care after being tracked through a phone call as through home visit (73% vs. 64%; p = 0.08). Of the 42% who self-reported missing scheduled clinic visits because of having travelled away from home, most (67%) were women. Forty-three percent reported socio-structural barriers to retention in care including forgetting the scheduled appointment, long distance to clinic, tight work schedules, stigma, ART side effects, having adequate supplies of drugs, and lack of food. The remaining 15% were unreachable or not available when tracked by phone and/or home visit.

In multivariable analyses, women were more likely to return to care after active tracking than men (adjusted risk ratio [ARR] 1.23; 95% CI 1.05–1.43; p = 0.009). Compared with persons who had travelled, those who were unavailable/unreachable at time of contact (ARR 0.85; 95% CI 0.75–0.95; p = 0.01) and those that reported other reasons for missing their scheduled appointments e.g., long distance to clinic, stigma, still having drug supplies or forgetting the appointment (ARR 0.41; 95% CI 0.28–0.59; p < 0.001) were less likely to return to care after missing a scheduled visit. Age, ART status and tracking method were not related to return to care (p = 0.27, 0.12 and 0.83, respectively).

### Discussion

In this retrospective cohort study of HIV-infected Ugandan adults, in which PLHIV who missed scheduled visits were actively followed through phone contact and/or home visits, most returned to care after being tracked. Female gender and prior receipt of ART were predictors of return to HIV care. Those who reported non-travel reasons for missed visits, or were unreachable by designated personnel, were significantly less likely to be retained in HIV care.

We found that women and PLHIV on ART were more likely to return to HIV care after missing a scheduled clinic visit. This finding is in agreement with prior studies in which gender and ART status were associated with return to care after tracking [16, 20–22]. In a systematic review of 42 studies from 12 countries, factors associated with lower retention rates included younger age, male gender, stigma, non-disclosure of HIV status, fear of drug side effects, and transport costs [23]. Other studies have found that denial of HIV status, healthy status perception, substance use, mental illness, distrust of medical providers, and side effects of HIV medications were associated with poorer retention [14, 23, 24]. Addressing these factors could increase the likelihood of staying engaged and retained in HIV care. In our study, a significantly higher proportion of men did not re-engage in care after active tracking. In East and Southern Africa, ART coverage is lower among men than women (57% vs 72%) [25], and men are less likely than women to adhere to HIV treatment resulting in poorer outcomes [26, 27]. Cultural constructs of masculinity and HIV stigma appear to influence health seeking behavior and compromise HIV care utilization in this setting [27]. HIV providers should utilize culturally appropriate initiatives to encourage health-enabling masculinities and support retention in care.

The proportion of PLHIV who ever missed a scheduled clinic visit in our study is similar to that reported in other studies of mobile populations [15, 16, 22]. Some PLHIV may have cycled in and out of care, given the mobile nature of the study population [9]. We observed that most PLHIV who were traced re-engaged in HIV care. Active tracing and linkage to care likely account for these findings. Monitoring retention on ART is key to estimating the proportion disengaging from care and developing targeted interventions to improve engagement in care, decreasing mortality, and minimizing adverse treatment outcomes [11]. Ancillary services such as case management, outreach, support groups, and patient navigation could further improve retention in care and facilitate attainment of national and global HIV treatment goals [28–33].

In our study, clinical, structural and psychosocial barriers to retention in care included long distance travel to clinic, tight work schedules, stigma, ART side effects, forgetting the scheduled appointment and food insecurity. This study was undertaken in Karamoja region. The region has poorer health outcomes than elsewhere in Uganda due to a combination of environmental, geographical, economic and cultural issues leading to conflict, food insecurity and malnutrition, high alcohol rates, gender based violence and low education rates [34]. Despite these difficulties, our findings are in striking agreement with other work from sub-Saharan Africa in which 65% reported structural barriers (transportation, distance to clinic, poverty), 33% reported clinic-based barriers (long waiting times), and 27% reported psychosocial barriers (social support, stigma and non-disclosure of HIV status) to retention in care [35]. These socio-structural factors are major determinants of retention in HIV care [36]. Accessing HIV care closer to home may decrease the cost and disruption of ART non-adherence. Community-based ART delivery programs, in which ART is delivered to consenting, stable participants at community drug distribution points, improve access to HIV treatment services, ART adherence and retention in care in resource-limited settings [37]. This alternative delivery model provides drug refills every 2–3 months, decreases waiting times, reduces travel time and costs, and provides psychosocial support by expert clients, thereby reducing the impact of socio-structural barriers on retention in care [37]. Community drug distribution points are an effective alternative delivery model which should be scaled up to improve retention in care in resource-limited settings.

### Conclusion

In conclusion, we found that nearly half of HIV-infected adults attending a rural HIV clinic in a region with poor health outcomes had ever missed a scheduled visit. Seven-in-ten PLHIV returned to HIV care following active tracing using home visits and phone calls. These data support active monitoring of patient retention in ART care and highlight the need for targeted interventions to engage men and others who struggle to maintain engagement in care especially in challenging environments.

## Limitations

Our study has limitations. We did not collect mortality data and those who returned to care may have been more likely to be healthy. Data were collected from a public sector clinic and there were considerable missing data. We did not evaluate socio-economic variables including level of education, occupation, marital status and disclosure of HIV status because these data are not routinely collected. Finally, we did not document CD4 counts, HIV viral load, and ART regimen to objectively assess HIV treatment outcomes after return to care. These factors may limit the generalizability of our findings. Nevertheless, we demonstrated that a substantial proportion of PLHIV re-engaged in HIV care following active tracing in a rural, resource-limited ART clinic.

## Abbreviations

HIV: human immunodeficiency virus
PLHIV: people living with human immunodeficiency virus
ARR: adjusted risk ratio
CI: confidence interval
ART: antiretroviral therapy
UNAIDS: United Nations Programme on HIV/AIDS
RRF: Regional Referral Hospital
CSF: Civil Society Fund
